# Muscle Atrophy Reversed by Growth Factor Activation of Satellite Cells in a Mouse Muscle Atrophy Model

**DOI:** 10.1371/journal.pone.0100594

**Published:** 2014-06-25

**Authors:** Simon Hauerslev, John Vissing, Thomas O. Krag

**Affiliations:** Neuromuscular Research Unit, Department of Neurology, Rigshospitalet, University of Copenhagen, Copenhagen, Denmark; University of Rome La Sapienza, Italy

## Abstract

Muscular dystrophies comprise a large group of inherited disorders that lead to progressive muscle wasting. We wanted to investigate if targeting satellite cells can enhance muscle regeneration and thus increase muscle mass. We treated mice with hepatocyte growth factor and leukemia inhibitory factor under three conditions: normoxia, hypoxia and during myostatin deficiency. We found that hepatocyte growth factor treatment led to activation of the Akt/mTOR/p70S6K protein synthesis pathway, up-regulation of the myognic transcription factors MyoD and myogenin, and subsequently the negative growth control factor, myostatin and atrophy markers MAFbx and MuRF1. Hypoxia-induced atrophy was partially restored by hepatocyte growth factor combined with leukemia inhibitory factor treatment. Dividing satellite cells were three-fold increased in the treatment group compared to control. Finally, we demonstrated that myostatin regulates satellite cell activation and myogenesis *in vivo* following treatment, consistent with previous findings *in vitro*. Our results suggest, not only a novel *in vivo* pharmacological treatment directed specifically at activating the satellite cells, but also a myostatin dependent mechanism that may contribute to the progressive muscle wasting seen in severely affected patients with muscular dystrophy and significant on-going regeneration. This treatment could potentially be applied to many conditions that feature muscle wasting to increase muscle bulk and strength.

## Introduction

**Figure 1:**
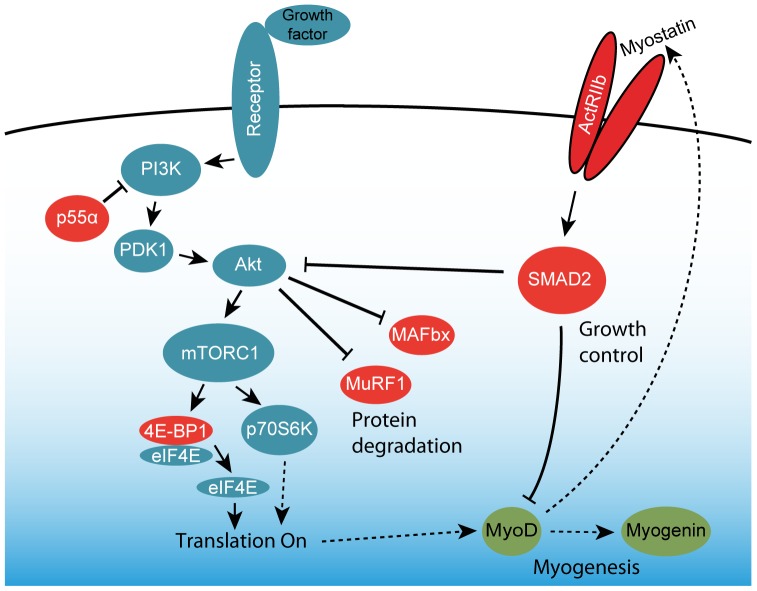
Model of signaling pathways involved in muscle regeneration. The satellite cell activating protein synthesis signaling (blue) and the interplay between myogenesis signaling (green) and myostatin-mediated growth control and protein degradation (red) which may form a negative feed-back loop to control growth or regeneration.

Muscular dystrophies comprise a large group of inherited disorders that lead to progressive muscle wasting. Much effort has been put into finding a cure or a treatment that alleviates the deficient motor function in these patients. Despite these efforts, no effective cure has yet been found against any of the muscular dystrophies, although viral delivery of the gene at issue and antisense oligonucleotide-mediated exon skipping have been attempted for many years. While such therapies can be adapted to treat a variety of different muscular dystrophies, these approaches are limited by technical issues related to 1) problems in delivering the gene to all affected cells and 2) development of an immune response against the protein or the vector harboring the gene for the missing protein, if the patient is completely devoid of the protein product in question. If possible, an approach that uses a compound known to the immune system of the patient would be preferable. Several approaches aimed at generating a larger muscle mass have been reported in the past decade, among them IGF-1 therapies and myostatin-inhibition [1], [2]. However, there are potentially other ways to induce hypertrophy or muscle repair. This could be accomplished by utilizing an already existing and highly efficient repair mechanism, the satellite cells, which are muscle specific stem cells. The satellite cells are quiescent in normal adult muscle, but activated in response to diverse stimuli such as injury, denervation, exercise, or stretching [3]. The mechanisms of satellite cell activation, proliferation, and migration have been well examined. HGF is the only known factor that is capable of activating satellite cells through the c-Met receptor [4], [5]. Activation of satellite cells lead to myoblast proliferation and terminal differentiation and fusion [6], [7]. HGF is present in uninjured myofibers and upon muscle injury may be secreted from them to the extracellular matrix surrounding myofibers and quickly acts on the satellite cell pool [5]. After satellite cell activation, expression of muscle regulatory factors, Myf5 and MyoD occurs, and at the beginning of differentiation, myogenin and MRF4 are expressed [8]. When HGF binds to the c-Met receptor tyrosine kinase, its cytoplasmic Tyr residues are autophosphorylated and bind the scaffolding adaptor protein Gab1, which leads to the activation of phosphatidylinositol 3-kinase (PI3K) and Ras-ERK mitogen-activated protein kinase cascade (Fig. 1) [9]. This suggests that controlled activation of c-Met signaling can be exploited in regenerative medicine. HGF is a well-known activator of satellite cells through the c-Met receptor, and there have been numerous *in vitro* experiments confirming that it activates satellite cells [5], [10]. However, it has also been reported that HGF delivered by intra-muscular injections inhibits differentiation in mice, which had been subjected to freeze injury and that repeated injections of HGF inhibited regeneration. This could be a matter of timing, as new injections may not have any effect before the effect of the preceding HGF injection had subsided. The choice of delivery method may also lead to a too high local concentration of HGF in the needle tract, leading to saturation of available receptors and ultimately premature initiation of a negative feed-back signal. This could render satellite cells insensitive to further HGF injections unless the time between injections was increased [4]. When designing our protocol, we took this into consideration, and used intraperitoneal injections rather than intra-muscular, which may yield a more even concentration of exogenous HGF in the muscles.

Leukemia inhibitory factor (LIF) is known to stimulate myoblast growth and fusion *in vitro* by receptor-mediated mechanisms [11], [12]. LIF acts directly at the site of injury and stimulates hypertrophy of the muscle fibers rather than hyperplasia [11]–[13]. In this study, we demonstrate that HGF-mediated activation of the satellite cells in hypoxia-induced muscular atrophy combined with LIF can reverse the loss of muscle mass. Rather than using normal mice under normoxic conditions, we use a hypoxia-induced atrophy model to enhance the effect of the treatment, since satellite cells are more prone to become activated under hypoxic compared to normoxic conditions [14], [15].

## Materials and Methods

### Animals

Only male mice were used for this study. C57BL/10ScSn bred at the animal facility at the University of Copenhagen and Mstn^ln/ln^ mice (strain #9345) were purchased from Jax mice. Control and treated mice were housed in separate cages (a maximum of 6 animals per cage) containing activity stimulating toys, at a 12∶12-h light-dark cycle and 22°C ambient temperature and received rodent chow (Lab Diet 5001) and water ad libitum. All mice included in this study were aged 15–16 weeks at the beginning of experiments. To perform the time-course study under normoxia following a single HGF injection, twenty-three C57BL/10ScSn mice were included. A second cohort of twenty-three C57BL/10ScSn mice were randomly assigned to a single HGF-treated group (n = 11 mice) or a PBS-treatment control group (n = 12 mice), and were exposed to 17 days of hypoxia. A third cohort of twenty-seven C57BL/10ScSn mice were randomly assigned to multiple treatments with HGF/LIF (n = 13 mice) or PBS-treated control group (n = 14 mice) and were exposed to 4 weeks of hypoxia. Finally, 16 Mstn ^ln/ln^ mice (strain #9345, purchased from Jax mice) and 16 C57BL/10ScSn mice were studied under normoxic conditions and received same treatment as the hypoxic mice.

### Ethical statement

This study was carried out in strict accordance with Danish law and all experiments were approved by the Danish Animal Experiments Inspectorate (Permit number 2012-15-2934-00595). All efforts were made to minimize suffering.

### Hypoxic protocol

In order to create a muscle atrophy model, we let the mice stay in a chamber with a gradually more hypoxic atmosphere starting at 12% until it reaches approx. 7.5% at day 7 simulating an ascent to an altitude of approximately 8,200 m above sea level (calculated from http://www.altitude.org). At day 2 the oxygen level was reduced from 12% to 10%, at day 4 further to 9% and at day 6 to 8%, by reducing the oxygen and increasing the nitrogen flow using flow meters to mix the gas, which entered the chamber at 3.0 l/min and exited through an one-way valve. The custom made normobaric hypoxic chamber had a volume of 85 litres and was maintained at 22°C and 45% humidity. Oxygen, carbon dioxide, temperature and humidity levels were monitored using sensors and a data logger (ScienceScope, Radstock, UK). To allow for visual inspection of health and condition of the mice during the light cycle, we installed a 360°/180°web-cam with a high-power zoom (BB-HMC581CE, Panasonic, Osaka, Japan) that could be remotely controlled by a PC or smartphone. Mice were briefly removed from the hypoxic chamber every second day so that cage bedding could be changed, water and chow consumption measured, and mice could be weighed and receive injections.

After two weeks of hypoxic exposure, treatment started with recombinant mouse HGF (2207HG/CF, R&D Systems, Minneapolis, MN) 20 ng/g body weight, followed 2 days after by recombinant mouse leukemia inhibitory factor (L5158, Sigma-Aldrich, St Louis, MO) 10 ng/g body weight by intraperitoneal injections for systemic delivery. Each injection contained BrdU (B5002, Sigma-Aldrich, St. Louis, MO) 10 ng/g body weight. (Mice were euthanized by cervical dislocation, and tibialis anterior (TA) and extensor digitorum longus (EDL) muscles of the lower limb were dissected. Muscles were trimmed of excess connective tissue, blotted dry, and weighed and then immediately flash frozen in isopentane cooled in liquid nitrogen and stored at −80°C.

### Immunohistochemistry and Histology

Cryo-sections of EDL were cut every 1 mm to determine the midbelly cross-sectional area of the EDL muscle. Sections were stained with hematoxylin and eosin for general histopathological evaluation, cross-sectional area and assessment of internally nucleated fibers (INF). For immunohistochemistry sections were fixed in 10% normal buffered formalin and subsequently blocked in buffer (5% normal goat serum in PBS) prior to staining. To assess the number of satellite cells undergoing divisions, sections were incubated with Pax7 (DSHB) diluted 1∶100 and Ki67 (#15580, Abcam, Cambridge, UK) antibodies diluted 1∶500. Positive nuclei were confirmed by DAPI nuclear stain (Invitrogen, Carlsbad, CA) and to be in a satellite cell position under the basal lamina by using an antibody against laminin (L9393; Sigma-Aldrich, St Louis, MO). To assess the number of cells with DNA synthesis since beginning of treatment, sections were incubated with BrdU antibody (clone G3G4, DSHB) diluted 1∶500 and DAPI nuclear stain (Invitrogen, Carlsbad, CA). Active regenerating myofibres were visualized using embyonic myosin heavy chain (F1.652, Vector Laboratories, Burlingame, CA) diluted 1∶100. Alexa 488 and 594 (Invitrogen, Carlsbad, CA) secondary anti- mouse and anti-goat antibodies were used at a 1∶500 dilution in PBS buffer. The sections were observed under a Nikon 80i microscope with epi-fluorescence. Area of EDL was measured using NIS-Elements Basic Research software (Nikon Instruments, Melville, NY).

### MR scanning

Whole body composition was analyzed without anesthesia or restraint by an EchoMRI 4-in-1 scanner (EchoMRI, Houston, TX) before and after 4 wk exposure to hypoxia. The scanner assessed fat mass, lean tissue mass, free fluids, and total body water content as independent characteristics. By freeze-drying the tibialis anterior muscle, water content was calculated during normoxia and after the hypoxic protocol.

### RT-qPCR

Muscle samples were collected over a six month period and stored at −80°C. RNA from TA muscles was extracted using TriZol i biologically duplicates and resuspended in RNA storage solution (AM7000, Invitrogen, Carlsbad, CA). All primers (Sigma-Aldrich, St Louis, MO) in this study were designed for unique transcripts using Primer-Blast (http://www.ncbi.nlm.nih.gov), to produce amplicons of 70–150 base pairs and to be exon-spanning (see Table S1 for primers). DNase I treatment of samples was therefore omitted. UNAFold 3.8 software was used to analyze the primers for potential secondary structures that may prevent efficient amplification. Primers were tested for efficiency (E) by serial dilution of cDNAs and primers were only accepted for use if 1.9≤E≤2.1. Purity of RNA samples with respect to protein and phenol contamination and concentration was assessed using a spectrophotometer (NanoDrop 2000, Thermo Scientific, Waltham, MA), and all samples had an OD260/280 of 1.8–2.0, indicating that purity of mRNA was acceptable. RNA integrity was analyzed using the automated microfluidics-based electrophoresis system 2100 Bioanalyzer (Agilent Technologies, Santa Clara, CA). All RNA samples had RIN values above 7 (mean 8.5±0.7) and a 28S/18S ratio between 1 and 2 indicative of an intact RNA sample. 500 ng RNA were converted into cDNA by reverse transcription using iScript (Bio-Rad, Hercules, CA).

Twenty µl reactions with SsoFast EvaGreen master mix (Bio-Rad, Hercules, CA) were amplified using a CFX96 RT-qPCR instrument (Bio-Rad, Hercules, CA). The melt curve analysis always displayed a single sharp peak thus confirming specificity of primer annealing and by running an agarose gel confirming the expected amplicon size. An inter-run calibrator, a no template control (NTC) reaction and a no reverse transcriptase (NRC) reaction were included in every run. NTC and NRC reactions were always below Cq<38. Results were analyzed and reference genes chosen using qBase Plus 2.0 software (Biogazelle, Zwijnaarde, Belgium).

### Western blotting

TA muscles were homogenized in ice-cold lysis buffer with protease and phosphatase inhibitors (10 mM Tris, pH 7.4, 0.1% Triton-X 100, 0.5% sodium deoxycholate, 0.07 U/ml aprotinin, 20 µM leupeptin, 20 µM pepstatin, 1 mM phenylmethanesulfonyl fluoride (PMSF), 1 mM EDTA, 1 mM EGTA, 1 mM DTT, 5 mM β-glycerophosphate, 1 mM sodium fluoride, 1.15 mM sodium molybdate, 2 mM sodium pyrophosphate decahydrate, 1 mM sodium orthovanadate, 4 mM sodium tartrate, 2 mM imidazole, 10 nM calyculin, 5 µM cantharidin) using a bead-mill at 4°C. Supernatants were collected and protein concentrations were determined using the Bradford assay. Equal amounts of extracted muscle proteins were separated on 10% TGX polyacrylamide gels (Bio-Rad, Hercules, CA) at 200V for 30 min. Proteins were transferred to PVDF membranes and post transfer membranes were stained with Sypro Ruby (Sigma-Aldrich, St Louis, MO) to ensure equal protein transfer. Membranes were blocked in Baileys Irish Cream (Dublin, Ireland) for 30 min and incubated overnight with primary antibodies (4E-BP1 (Thr37/46); Akt (Ser473); Akt; eIF4E (Ser209); mTOR (Ser2448); p70S6K (Thr389); p70S6K; PDK1 (Ser241); PI3K (Tyr458(p85)/Tyr199(p55)); Ubiquitin (P4D1) from Cell Signaling Technologies, Danvers, MA. α-Tubulin (12G10) and Myogenin (F5D), (Developmental Studies Hybridoma Bank, Iowa City, IA). Myostatin (AB3239, Millipore, Billerica, MA), MAFbx and MuRF1, (Sigma-Aldrich, St Louis, MO), MyoD1 (5.8A, Vector Laboratories, Burlingame, CA.). Secondary antibodies coupled with horseradish peroxidase diluted 1∶10000 were used to detect primary antibodies (DAKO, Denmark). Immuno-reactive bands were detected using a SuperSignal West Dura kit (Thermo Scientific, Waltham, MA), quantified using a GBox XT16 darkroom and GeneTools software (Syngene, UK) was used to measure the intensities of immune-reactive bands on 16-bit digital photos. Immuno-reactive band intensities were normalized to the intensity of the α-tubulin bands for each subject to correct for differences in total muscle protein loaded on the gel. For Akt(Ser473) and p70S6K (Thr389) these targets were calculated and shown as ratios of total Akt and p70S6K respectively.

### Statistics

One-way ANOVA with Bonferroni *post hoc* correction for multiple comparisons was used to assess differences among groups for each dependent variable. Student's t-test was used to test for significant difference between treated animals and controls. A p-value below 0.05 was considered significant.

## Results

### Protein synthesis and myogenic signaling following HGF treatment of normoxic mice

**Figure 2:**
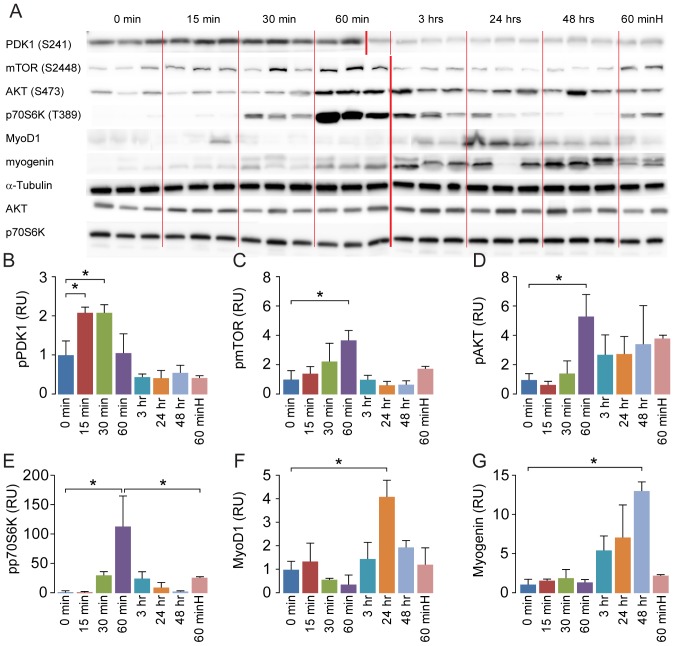
Hepatocyte growth factor activates protein synthesis pathway and increases protein expression of myogenic factors. In order to establish if HGF initiated the protein synthesis and myogenesis pathways and the timeframe in vivo, we injected a total of 21 normoxic mice (N = 3 for each time point) with hepatocyte growth factor (20 ng HGF/g body weight) and sacrificed them at time points between 0 and 48 h. A western blot of components of the protein synthesis and myogenesis pathways demonstrates how the signal activates the different proteins with time and eventually leads to an increased expression of myogenic factors (A). Two mice were given a 10 times higher dose of hepatocyte growth factor (200 ng HGF/g body weight) and sacrificed after 60 min ( = 60 minH). Western blots of tibialis anterior homogenates (N = 3 for each time point) shows activation of protein synthesis pathway through phosphorylation of PDK1 (B)/mTOR (C)/Akt (D)/p70S6K (E), and increased protein expression of myogenic factors MyoD (F) and myogenin (G). A 200 ng HGF/g body weight dose of hepatocyte growth factor yields a diminished response compared to a 20 ng HGF/g body weight dose RU = Relative Units. Error bars are SD. Thin dividing lines on the blot are for clarification only whereas the bold red line symbolizes that samples were run on two separate gels. One-way ANOVA with Bonferroni *post hoc* correction for multiple comparisons was used to assess differences among groups for each dependent variable. *denotes significant (*P*<0.05). Data are representative of one independent experiment.

**Figure 3:**
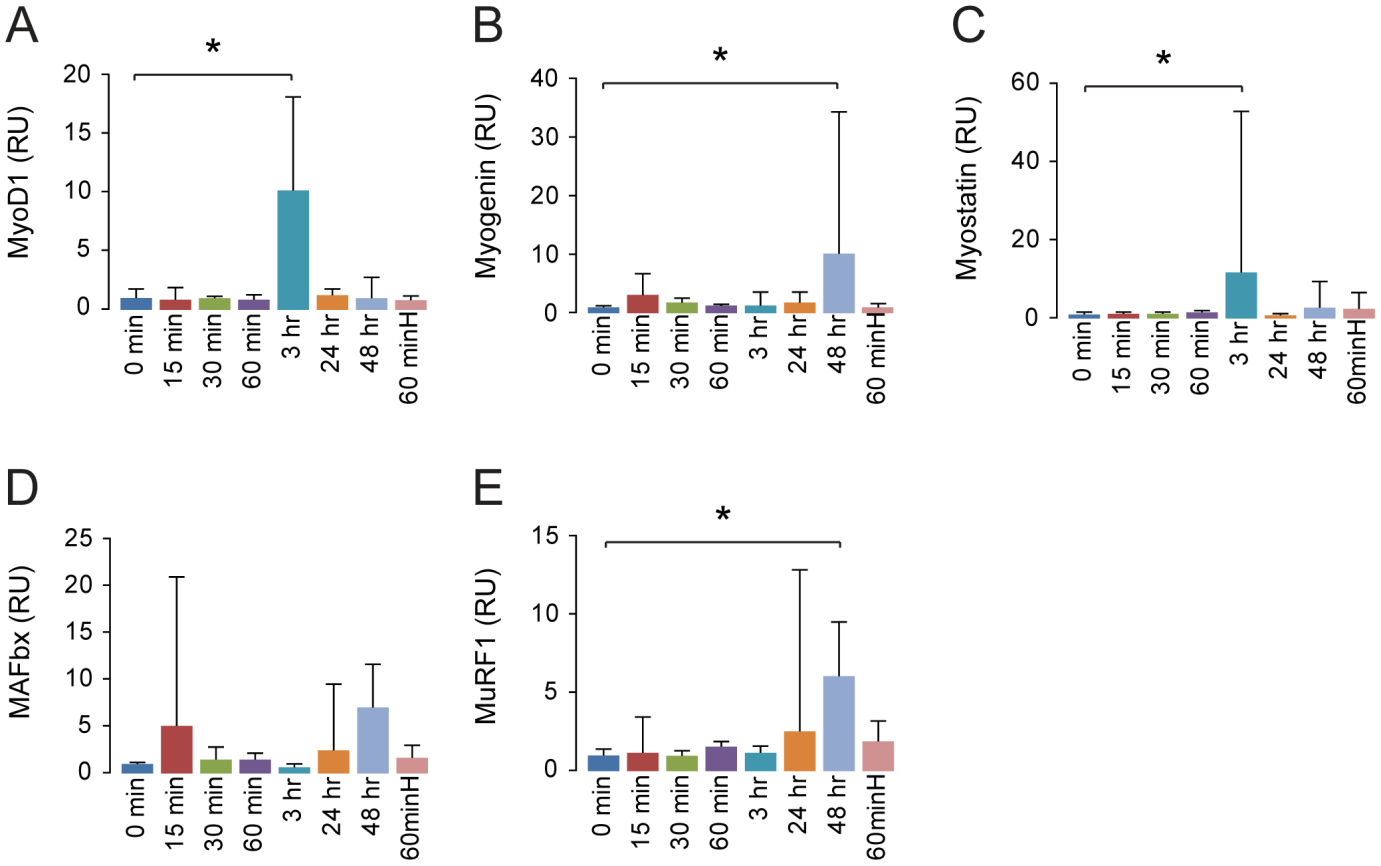
Hepatocyte growth factor increases mRNA expression of myogenic factors and myostatin. Total RNA was purified from the same samples as used in figure 2. RT-qPCR analysis of mRNA expression levels in tibialis anterior of shows hepatocyte growth factor increases myogenic factors MyoD, myogenin (A,B). Muscle negative regulation and protein breakdown pathway myostatin, MAFbx and MuRF1 (C-E) were increased following a single injection with hepatocyte growth factor. RU = Relative Units. N = 3 for all time points, except for 60 minH ( = a 10 times higher dose of hepatocyte growth factor [200 ng HGF/g body weight] sacrificed after 60 min) where N = 2. Error bars are SD; One-way ANOVA with Bonferroni *post hoc* correction for multiple comparisons was used to assess differences among groups for each dependent variable. *denotes significant (*P*<0.05). Data are representative of four independent experiments.

To investigate the mechanistic effect of treatment with HGF we first performed a 0-48-hour time-course study in normoxic mice. Following a single injection with HGF, we found an increased activity of the Akt/mTOR/p70S6K pathway involved in protein synthesis within the first hour, and an elevation in myogenic factors MyoD and myogenin after 24–48 hours (p<0.05) (Fig. 2). To test if the response to HGF could be enhanced by increasing the dose, we treated animals with a 10 times higher dose (200 ng HGF/g body weight), and the mice were then sacrificed after one hour. We found a reduced response in activation of p70S6K compared to the low dose group at the same time point (Fig. 2E). Performing RT-qPCR analysis on the time-course samples, we found that the elevation of protein expression of myogenin was mirrored in the mRNA response (Fig. 3). MyoD and myostatin mRNA were increased after 3 hours, while MuRF1 was increased after 48 hours.

### HGF/LIF treatment of hypoxia-induced muscle atrophy leads to increased muscle mass

**Figure 4:**
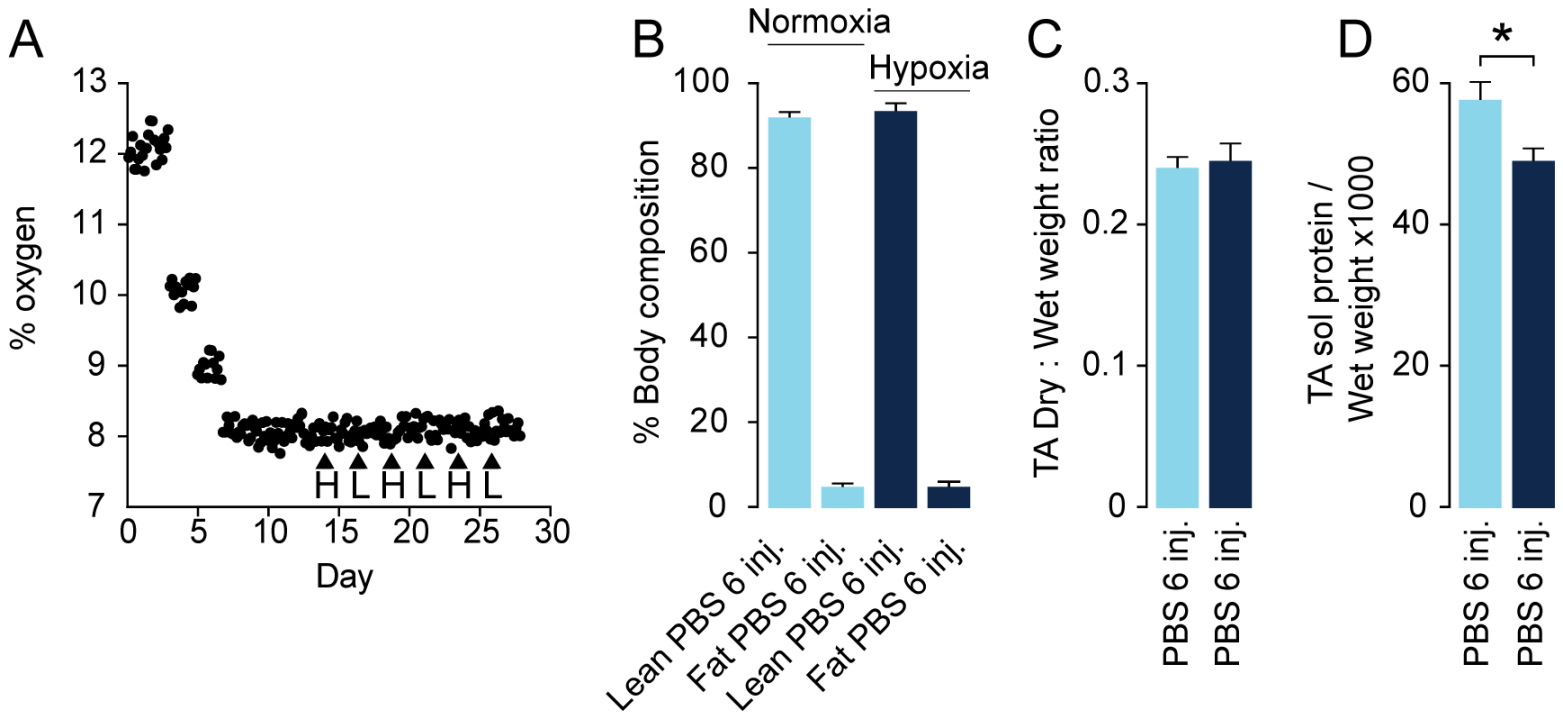
The hypoxic protocol and verification of atrophy model. In order to create a muscle atrophy model, we let the mice stay in a chamber with a gradually more hypoxic atmosphere until it reaches approx. 7.5%. Oxygen content in the hypoxic chamber was monitored during the hypoxic protocol (•) and time points for alternating intraperitoneal injections (▴) with hepatocyte growth factor = H and leukemia inhibitory factor = L (A). Hypoxia did not change body composition (Lean/fat) measured by quantitative MRI of PBS-injected normoxic mice (N = 8, light blue), PBS-injected hypoxic mice (N = 8, dark blue) (B). Hypoxia did not change muscle water content based on dry weight: wet weight ratio in tibialis anterior (TA) of PBS injected normoxic mice (N = 8, light blue), PBS injected hypoxic mice (N = 8, dark blue) (C). Hypoxia induced loss of muscle protein measured as total soluble protein in muscle homogenates per wet weight of PBS-injected normoxic mice N = 8 (light blue) and PBS injected hypoxic mice (N = 8, dark blue) (D). Error bars are SD; Statistical significance was determined by a two-tailed Student's t-test. *denotes significant (*P*<0.05). Data are representative of one independent experiment.

**Figure 5:**
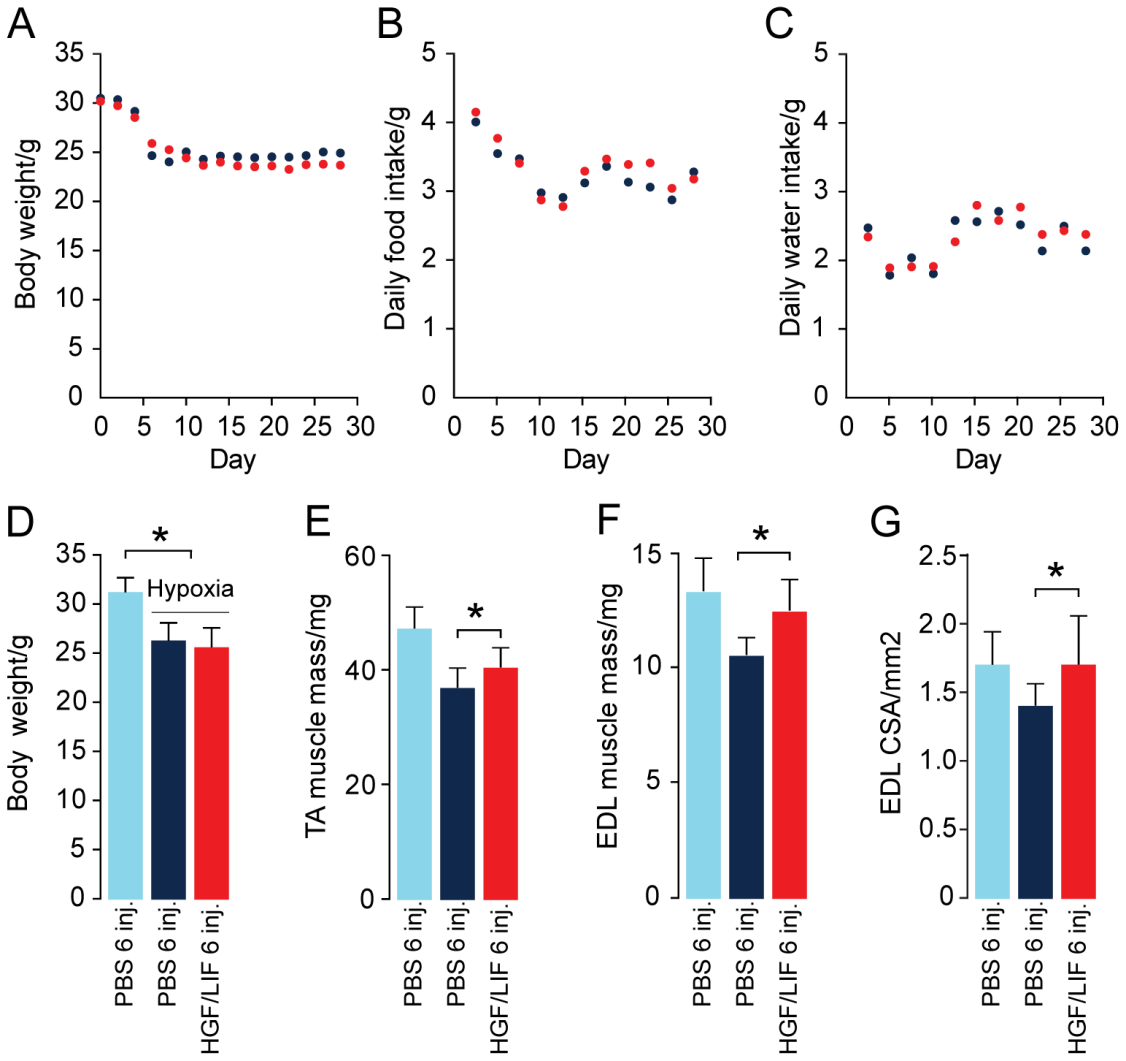
Effect of alternating hepatocyte growth factor and leukemia inhibitory factor treatment on bodyweight and muscle mass during hypoxia. Hypoxia induced loss of mean bodyweight of both control PBS injected mice group (N = 14, dark blue) and HGF/LIF treated hypoxic mice group (N = 13, red) (A,D). PBS injected normoxic mice (N = 8, light blue) are included for comparison (D-G). Daily food and water intake per mouse during hypoxic protocol in the HGF/LIF treated group (N = 13, red) and in the control group (N = 14, dark blue) (B,C). Compared to PBS injected normoxic mice (N = 8, light blue) hypoxia induced loss of wet weight muscle mass of both tibialis anterior (TA) and extensor digitorum longus (EDL) (E,F). Alternating treatment of hypoxic mice with HGF/LIF (N = 13, red) increased muscle mass compared to PBS injected hypoxic mice (N = 14, dark blue) (E,F). Hypoxia induced loss of midbelly cross-sectional area of EDL of PBS injected hypoxic mice (N = 14, dark blue) compared PBS injected normoxic mice (N = 8, light blue). Alternating treatment of hypoxic mice with HGF/LIF increased cross-sectional area of EDL compared to PBS injected hypoxic mice (N = 14, dark blue) (G). Error bars are SD; Statistical significance was determined by a two-tailed Student's t–test. *denotes significant (*P*<0.05). Data are representative of one independent experiment.

**Figure 6:**
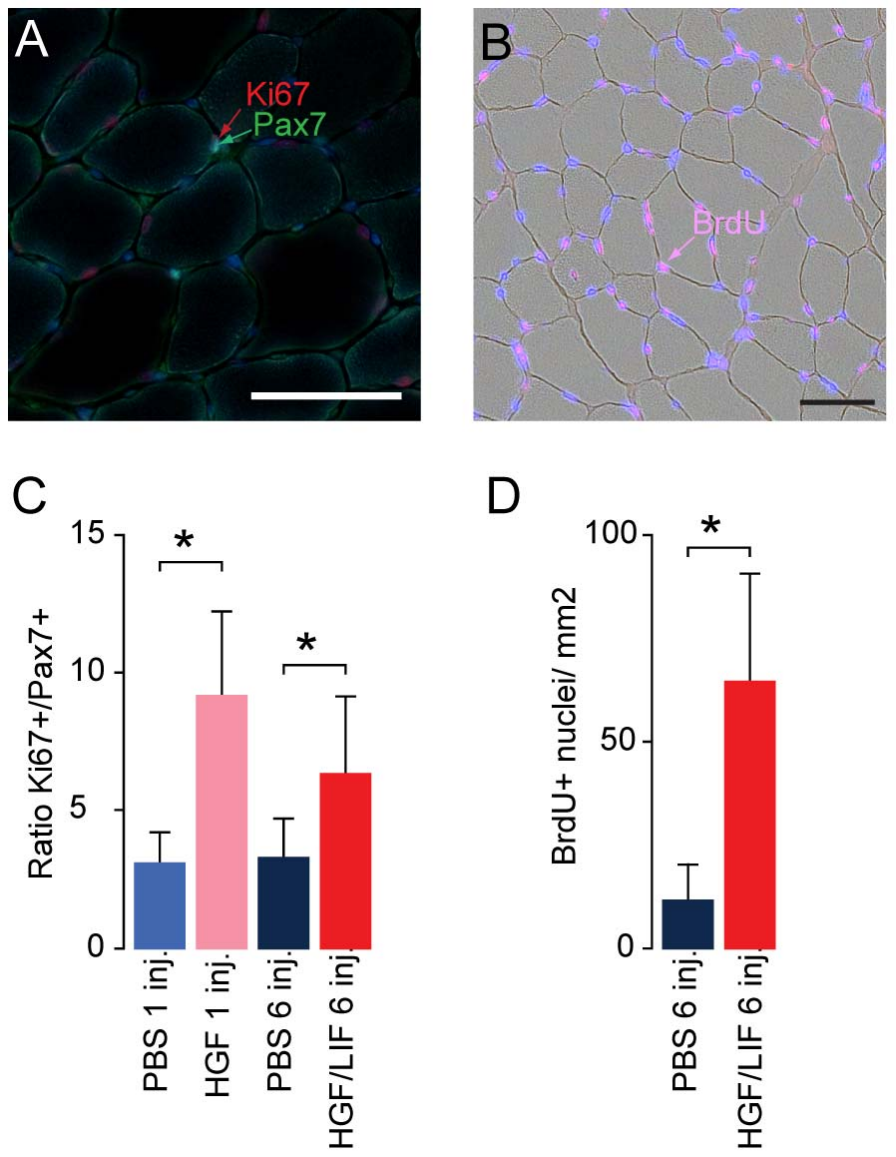
Hepatocyte growth factor and leukemia inhibitory factor treatment increases the mitosis of satellite cells in vivo during hypoxia. Tibialis anterior sections were incubated with antibodies specific for satellite cell marker Pax7, proliferation marker Ki67 and DNA synthesis marker BrdU. Treatment of hypoxic mice with hepatocyte growth factor alone or in combination with leukemia inhibitory factor increased ratio of satellite cells undergoing divisions in following one HGF injection (N = 11, pink) compared to PBS (N = 12, blue) and multiple HGF/LIF (N = 13, red) injections compared to multiple PBS (N = 14, dark blue) or (A,C). Treatment of hypoxic mice with hepatocyte growth factor in combination with leukemia inhibitory factor (N = 13, red) increased number of cells with incorporated BrdU following DNA synthesis in following compared to multiple injections with PBS (N = 14, dark blue) (B,D). Bar in picture is 50 µm. Error bars are SD; Statistical significance was determined by a two-tailed Student's t–test. *denotes significant (*P*<0.05). Data are representative of one experiment.

To investigate if this treatment could have an effect on muscle atrophy, we used hypoxia to induce muscle atrophy. B10 mice were exposed to 2 weeks of hypoxia before starting treatment. Hypoxia was continued during treatment with HGF/LIF (N = 13) or placebo (N = 14) (Fig. 4*A*). Quantitative MRI confirmed that body weight loss during the four weeks of exposure to hypoxia did not alter whole body composition (Fig. 4*B*). Subsequently, we determined muscle wet weight and then freeze-dried the tibialis anterior (TA) muscles in vacuum to investigate if muscle weight loss obtained during the four weeks of exposure to hypoxia was caused by altered muscle water content. Based on dry weight: wet weight ratio in TA in normoxia (0.24±0.01) and after the hypoxic protocol (0.25±0.01) muscle water content was unchanged (*P*<0.13) (Fig. 4*C*). We also found that hypoxia induced loss of muscle protein measured as total soluble protein in muscle homogenates per wet weight (*P*<0.03) (Fig. 4*D*). Exposure to hypoxia for four weeks resulted in a significant (*P*<0.01) 18% decrease in body weight (Fig. 5*A*). There was no significant difference in neither food nor water intake between groups (Fig. 5*B and C*). There was no significant difference in body weight between groups at the beginning of the experiment (31.4±1.5 and 31.6±2.2 g for PBS- and HGF/LIF-treated mice, respectively, *P*<0.83) or at the end (26.1±1.8 and 25.3±2.1 g for PBS- and HGF/LIF-treated mice, respectively, *P*<0.33) (Fig. 5*A and D*). Alternating treatment of atrophic mice with HGF and LIF significantly increased muscle mass of both TA (9%, *P*<0.02) and extensor digitorum longus (EDL) (18%, *P*<0.003) compared to control mice during exposure to hypoxia (Fig. 5*E and F*). Correspondingly, the cross-sectional area of EDL increased 22% (*P*<0.01) in the HGF/LIF treated group compared to hypoxic controls, and compared to normoxia, atrophy was reversed (*P*<0.99) in the HGF/LIF treated group (Fig. 5*G*). Satellite cells undergoing divisions were 3-fold increased in hypoxic mice treated with a single HGF injection (9%±3%) compared to hypoxic mice injected with PBS (3%±1%) (Fig. 6*A and C*). Repeated alternating injections with LIF/HGF increased the replicative response (59±28 BrdU+nuclei/mm^2^) compared to hypoxic mice injected with PBS (9±8 BrdU+nuclei/mm^2^) (*P*<0.01) (Fig. 6*B and D*).

**Figure 7:**
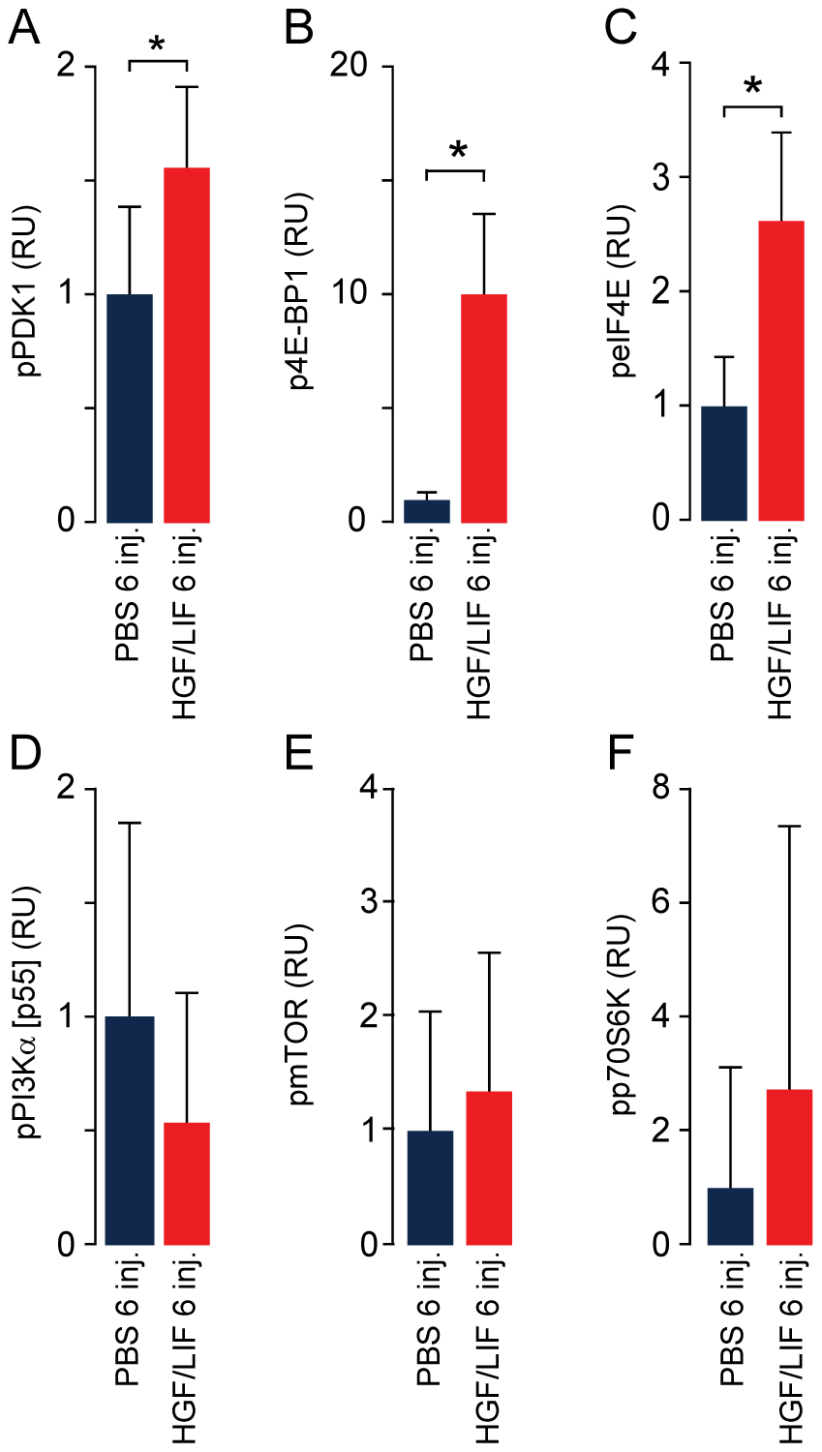
Effect of alternating hepatocyte growth factor and leukemia inhibitory factor treatment on protein synthesis pathway during hypoxia. Western blots of tibialis anterior homogenates from hypoxia induced atrophic mice shows activation of protein synthesis pathway after repeated alternating injections with hepatocyte growth factor and leukemia inhibitory factor (N = 13, red) compared to PBS (N = 14, dark blue). This is based on evaluation of the phosphorylation of components from the protein synthesis pathway; pPI3K [p55α] (A), pPDK1 (B), pmTOR (C), pp70S6K (D), p4E-BP1 (E) and peIF4E (F). RU = Relative Units. Error bars are SD; Statistical significance was determined by a two-tailed Student's t–test. *denotes significant (*P*<0.05). Data are representative of one experiment.

**Figure 8:**
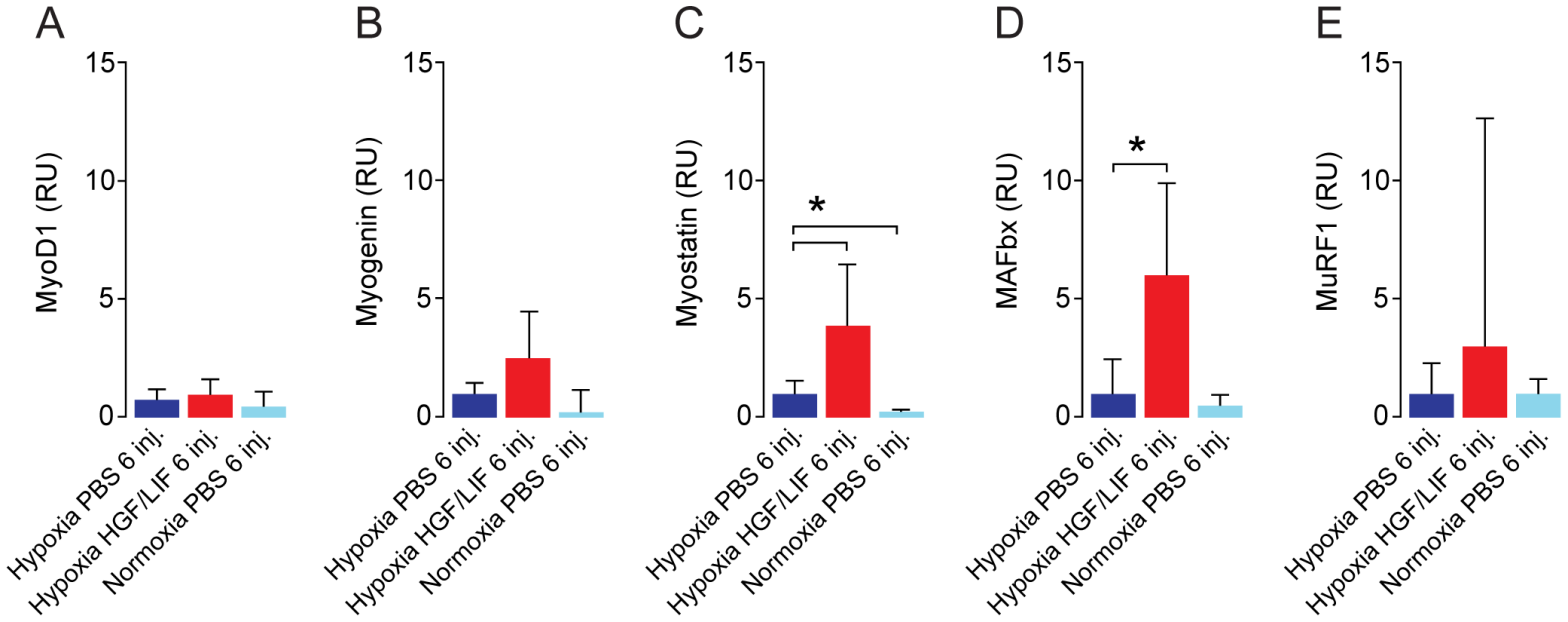
Alternating hepatocyte growth factor and leukemia inhibitory factor treatment increases mRNA expression of myostatin and MAFbx during hypoxia. Total RNA was purified from the same samples as used in figure 7. RT-qPCR analysis of mRNA expression levels in tibialis anterior of shows elevated mRNA expression levels of myostatin, MAFbx and (C,D) after repeated injections with alternating HGF/LIF (N = 13, red) in hypoxia induced atrophic mice compared to PBS (N = 14, dark blue). Normoxic PBS treated mice (N = 8, light blue) were included for comparison. mRNA expression levels of MyoD, myogenin and MuRF1 was unchanged by treatment (A,B,E). RU = Relative Units. Error bars are SD; One-way ANOVA with Bonferroni *post hoc* correction for multiple comparisons was used to assess differences among groups for each dependent variable. *denotes significant (*P*<0.05). Data are representative of four independent experiments.

Repeated alternating injections with LIF/HGF in hypoxic mice showed that these growth factors trigger the activation of factors involved in protein synthesis (PDK1, 4E-BP1 and eIF4E; Fig. 7*A,B and C*). The activation levels of other factors in this signaling pathway were not significantly changed (PI3Kα(p55), mTOR and p70S6K; Fig. 7*D,E and F*). mRNA levels for MyoD and myogenin were not significantly different between PBS and HGF/LIF treated hypoxic mice. However, the myostatin pathway for growth control as well as protein degradation in terms of MAFbx was significantly elevated in the HGF/LIF treated hypoxic mice (Fig. 8*C and D*). We found no significant difference in the mRNA level of MuRF1 in HGF/LIF treated mice versus controls (Fig. 8*E*).

### Myostatin represses HGF/LIF treatment in normoxic mice and controls protein degradation

**Figure 9:**
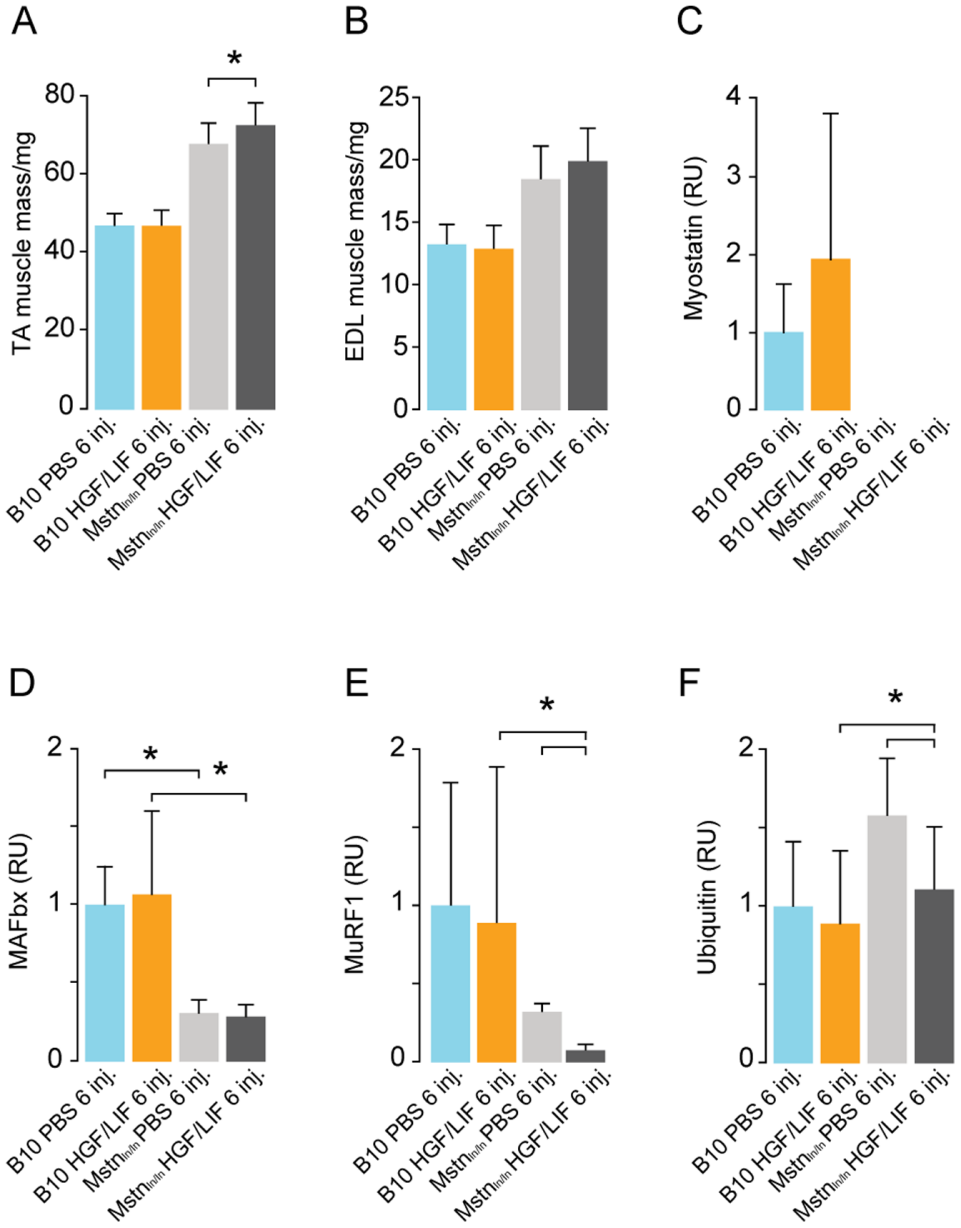
Alternating hepatocyte growth factor and leukemia inhibitory factor treatment increases muscle mass and decreases protein breakdown pathway in normoxic myostatin deficient mice. Alternating injections of normoxic B10 mice with hepatocyte growth factor and leukemia inhibitory factor (N = 8, yellow) did not change wet weight muscle mass of Tibialis anterior = TA and extensor digitorum longus = EDL (compared to PBS injected mice (N = 8, light blue) (A,B). Alternating injections of normoxic Mstn^ln/ln^ mice with hepatocyte growth factor and leukemia inhibitory factor increased TA muscle weight compared to PBS injected Mstn^ln/ln^ mice (N = 8, light grey). Western blot data of tibialis anterior homogenates (C-F) showed no change in muscle negative regulation and protein breakdown pathway (myostatin, MAFbx, MuRF1 and ubiquitin) after repeated alternating injections with HGF/LIF in normoxic B10. Repeated alternating injections with HGF/LIF in Mstn^ln/ln^ mice decreased expression of protein breakdown pathway MAFbx, MuRF1 and ubiquitin (D-F). RU = Relative Units. Statistical significance was determined by a two-tailed Student's t–test. *denotes significant (*P*<0.05). Data are representative of one independent experiment.

**Figure 10:**
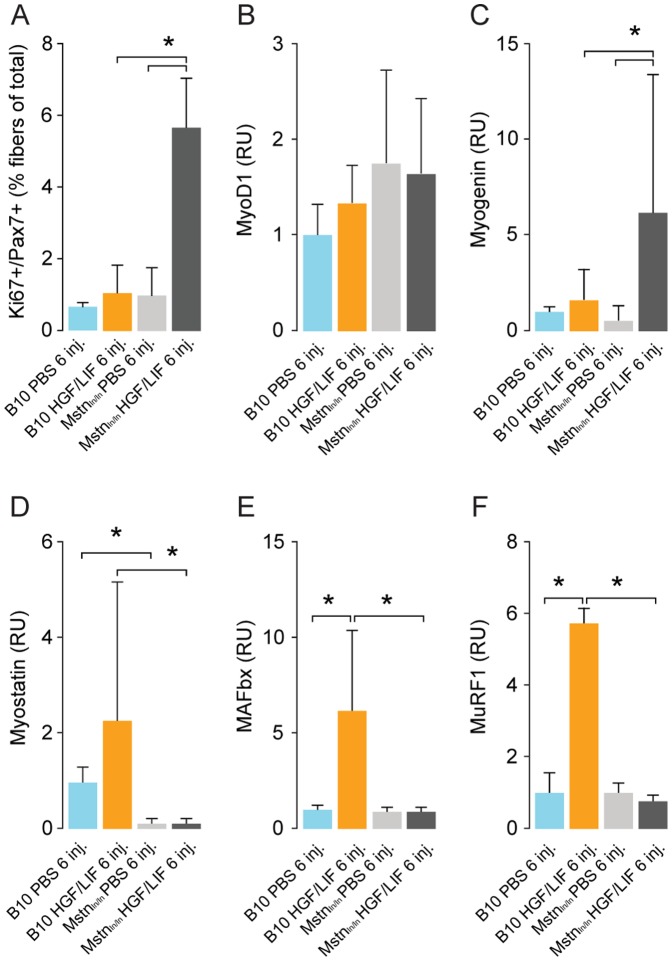
Alternating hepatocyte growth factor and leukemia inhibitory factor treatment increases satellite cells undergoing mitotic divisions and decreases mRNA expression levels of protein breakdown pathway components in normoxic myostatin deficient mice. Tibialis anterior sections were incubated with antibodies specific for satellite cell marker Pax7, proliferation marker Ki67. Alternating injections of normoxic B10 mice with hepatocyte growth factor and leukemia inhibitory factor (N = 8, yellow) did not change ratio of satellite cells undergoing mitotic divisions compared to PBS injected B10 mice (N = 8, light blue) (A). Ratio of satellite cells undergoing mitotic divisions was significantly increased in normoxic Mstn^ln/ln^ mice after repeated alternating injections with HGF/LIF (N = 8, dark grey) compared to PBS injected Mstn^ln/ln^ mice (N = 8, light grey). RT-qPCR analysis of mRNA expression levels yielded no difference in levels of MyoD was observed between groups (B). Alternating injections of normoxic Mstn^ln/ln^ mice with hepatocyte growth factor and leukemia inhibitory factor lead to increased levels of myogenin and decreased levels of myostatin, MAFbx and MuRF1 compared to treated normoxic B10 mice (B-F). RU = Relative Units. Error bars are SD; One-way ANOVA with Bonferroni *post hoc* correction for multiple comparisons was used to assess differences among groups for each dependent variable. *denotes significant (*P*<0.05). Data are representative of one independent experiment (A) and four independent experiments (B-F).

We wanted to determine if there was a mechanism linking myostatin to MAFbx- and MuRF1-related protein degradation *in vivo*. For this purpose, we carried out HGF/LIF treatment of Mstn^ln/ln^ and wild-type mice under normoxic conditions as with the time-course experiment since myostatin-mediated growth-repression is probably more active under normal conditions (Fig. 3). Muscle weight of both TA and EDL normoxic was increased 40% in Mstn^ln/ln^ compared to wild-type mice (Fig. 9*A and B*). Treatment with HGF/LIF resulted in 7% increase in TA muscle weight compared to control group in Mstn^ln/ln^ mice (*P*<0.04), but not in normoxic wild-type mice (Fig. 9*A*). While there was no significant difference in levels of myostatin in B10 mice treated with alternating injections of HGF/LIF compared to controls, we found that HGF/LIF in Mstn^ln/ln^ mice lead to a very significant down-regulation of MAFbx and MuRF1 (Fig. 9*C-E*). This is further supported by the significant decrease in ubiquitinylated proteins in the HGF/LIF treated Mstn^ln/ln^ mice compared to the PBS treated Mstn^ln/ln^ mice (*P*<0.03) (Fig. 9*F*). Treatment with HGF/LIF resulted in a 6-fold more mitotically active state in normoxic Mstn^ln/ln^ vs. wild-type mice satellite cells (Fig. 10*A*). Expression of MyoD mRNA showed no difference among groups, whereas myogenin mRNA increased 10-fold in treated Mstn^ln/ln^ mice compared to Mstn^ln/ln^ injected with PBS (Fig. 10*B and C*). Transcription of myogenin was 3-fold increased in treated Mstn^ln/ln^ mice compared to treated wild-type mice (Fig. 10*C*). Treating normoxic Mstn^ln/ln^ mice with HGF/LIF did not lead to negative growth control, unlike in wild-type normoxic mice, where the myostatin pathway for growth control appears induced, consistent with our observations during hypoxia (Fig. 10*D-F*).

## Discussion

Two decades following the cloning of the dystrophin gene, the gene involved in Duchenne muscular dystrophy (DMD), much effort has been put into finding a cure or therapy that alleviates symptoms. Generally speaking, these approaches can be divided into two categories; those that attempt to rectify the causative problem, i.e. targeting the dystrophin gene, and those that in a more general way aim to repair or replace damaged tissue by more unspecific stimulation of regeneration. In the present study, we have chosen a strategy that aims at balancing the degeneration – regeneration cycle, which in muscular dystrophies is skewed towards degeneration, thus leading to a progressive loss of muscle mass. In this study, we have demonstrated the following novel findings: 1. HGF treatment *in vivo* leads to up-regulation of the Akt/mTOR/p70S6K protein synthesis and hypertrophy pathway. 2. HGF/LIF can reverse atrophy through activation of the satellite cells. 3. The efficiency of the treatment appears to be inversely related to myostatin-dependent protein degradation. For patients who are suffering from muscle loss/wasting, in whom the regenerative capacity of muscles is unaffected, HGF/LIF and a myostatin-inhibitor could potentially be a new treatment that can boost muscle regeneration, and reverse muscle atrophy.

We wanted to investigate how HGF affects protein synthesis over time by treating mice with HGF under normoxic conditions. Our results demonstrate that HGF *in vivo* rapidly leads to activation of the protein synthesis pathway and subsequent up-regulation of MyoD and myogenin. Interestingly, treating mice with a 10 times higher dose of HGF resulted in a poorer activation of the protein synthesis pathway, suggesting that a high concentration of HGF may inhibit rather than activate the protein synthesis pathway, which has also been suggested recently [16]. A simpler explanation could be that HGF at the 20 ng/g bodyweight concentration we administered is sufficient to saturate the available c-met receptors of the quiescent satellite cells. After all, there are a finite number of quiescent satellite cells and it is debatable if all subpopulations of satellite cells respond in the same way to HGF. However, as the kinetics of HGF *in vivo* is not well understood this remains speculation. While the HGF time course results were encouraging, our qPCR data from the above experiment suggests that a down-stream effector of HGF activates a negative feedback mechanism acting through myostatin, which regulates growth negatively [16].

We demonstrated that HGF leads to an increase in MyoD mRNA 3 hours post-injection and a peak in MyoD protein after 24 hours. In line with the RNA expression, the protein expression levels of MyoD and myogenin also increased. Concurrently, at 3 hours, possibly later, but before 24 hours post-injection, myostatin mRNA peaked as well. Previous studies have established that MyoD is one of the key transcription factors regulating myostatin expression, presumably as a negative feed-back mechanism, and that myostatin subsequently is capable of repressing MyoD expression through SMAD3, part of the TGFβ-family/SMAD signaling pathway [17], [18]. Akt represses expression of the muscular atrophy inducing E3-ubiquitin ligases MAFbx and MuRF1 through phosphorylation of FoxO transcription factors [19] and conversely the repressive action of Akt on MAFbx and MuRF1 can be lifted by myostatin through SMAD2 [20], [21]. From 3 to 24 hours post-injection, Akt-activity decreased, presumably due to myostatin-mediated SMAD2/3 inhibition [20]. Consistent with this, we saw an increased expression of MAFbx and MuRF1 at 24 and 48 hours post-injection, confirming a previous *in vitro* study where HGF lead to myostatin up-regulation and increased MAFbx and MuRF1 expression 48 hours after injection of HGF [22]. The specific role of MAFbx and MuRF1 in this context is not fully known, but as MAFbx is suggested to target signaling pathways rather than structural components, and MuRF1 can interact with myosin heavy chain and the giant sarcomeric protein titin, it is possible that they down-regulate signaling pathways and remove temporary or immature elements no longer needed for the myogenic process to proceed [23]. The fact that myogenin mRNA and protein peaked at 48 hours post-injection is consistent with the above, since the need for proliferation may have abated and the myostatin-pathway has switched off further activation and proliferation leading to a cellular focus on terminal differentiation and fusion with the myofibers.

Under normal condition, the effect of HGF treatment on various morphological and physiological properties may be too subtle to allow proper analysis, unless the treatment is delivered over the course of several months. For this reason we chose to use a hypoxia-induced atrophy model to prove conceptually if treatment of atrophic/dystrophic muscle with HGF had any effect on activation of satellite cells and gain of muscle mass based on our data on increased HGF-induced protein synthesis. The use of an atrophy mouse model for evaluating the efficacy of a therapy has been described previously, in a study where treatment with soluble activin receptor IIb was demonstrated to counteract the hypoxia-induced atrophy of muscles [24].

We combined multiple HGF injections with LIF to enhance proliferation and differentiation of satellite cells. Due to the relatively short period of treatment, we did not expect and did not achieve any increased body weight in HGF/LIF treated hypoxic mice. However, it is clear that HGF/LIF leads to increased muscle mass as demonstrated in both TA and EDL. Our results also suggest that there may be an upper limit to the treatment efficiency as multiple HGF and LIF treatments did not lead to more mitotic satellite cells than could be achieved by one HGF injection, which is consistent with earlier findings [4]. Interestingly, we found no difference in the number of mitotic satellite cells when comparing the group that had one injection of HGF/LIF to the group that had received six injections. As the number of quiescent satellite cells are considered fairly constant, our results suggest, that either the administered HGF had activated a maximum of quiescent satellite cells or activated a negative feedback loop aborting the activity of more HGF within a limited period of time. There are likely some inherent limitations to the actions of HGF. It acts a switch, so that once the satellite cell is activated there is a turn-around time for the activated and dividing satellite cells before they enter the G_0_-phase again, expressing c-met receptors. In addition, HGF requires heparan-sulfate proteoglycans (HSPG) as co-receptors with the level of sulfation determining the activity of the growth factor [7]. The sulfation is regulated by Sulf1, a sulfatase, which itself becomes up-regulated as satellite cells become activated, presumably as an extracellular negative feed-back preventing an overextension of the regenerative system [25]. This means that additional HGF may not be useful even though the satellite cells have entered the G_0_-phase, as the Sulf1 must be down-regulated before proper sulfation of the HSPG can be attained as required by HGF. When this has happened, HGF may have cleared the body. The up-regulated myostatin may be an additional factor suppressing an increase in mitotic satellite cells in mice that had received multiple HGF/LIF injections, as myostatin not only effectively represses MyoD dependent activation of satellite cells, but also blocks proliferation by modulation of the cell cycle progression through p21 [26]. Hence, myostatin negatively regulates the G1 to S progression, and thus maintains the quiescent status of satellite cells. However, while up-regulation of p21 inhibits proliferation, it also promotes differentiation [27].

A positive change in activation of satellite cells and gain of muscle mass is considered beneficial for treating muscular dystrophies. After the HGF/LIF treatment, we found that cross-sectional area and weight of EDLs in HGF/LIF treated hypoxic animals were not significantly different from the EDLs of normoxic animals. In other words, the EDLs had recovered due to the treatment. This finding is supported by an increase in dividing satellite cells and parts of the protein synthesis signaling. However, as we saw no significant difference in the levels of MyoD and myogenin in between treated and untreated hypoxic animals and normoxic controls, we found that myostatin and MAFbx, but not MuRF1, were increased in the HGF/LIF treated hypoxic animals. Clearly there is a connection between satellite cell activation/proliferation through HGF, a rise in myostatin, MAFbx and subsequent suppression of MyoD and myogenin.

The myostatin signaling in the time-course treatment of mice with HGF as well as the increased level of myostatin in the HGF/LIF treated hypoxic mice lead us to investigate how myostatin affects HGF treatment. Consistent with our findings from the time-course experiment, we found that HGF/LIF-treated Mstn^ln/ln^ mice have a 5-fold higher level of mitotic satellite cells under normoxic conditions compared to PBS controls (C57BL/10 and Mstn^ln/ln^ mice) and C57BL/10 mice treated with HGF/LIF. It is evident from these observations that myostatin very effectively represses satellite cell activation. In addition, we found that the level of mitotic satellite cells is approximately 3 times higher in atrophic mice exposed to hypoxia compared to that in normoxic mice treated with just PBS (controls). This is supported by previous findings *in vitro* where a hypoxic environment had a positive effect on satellite cell proliferation and differentiation [14], [15]. Consistent with this, the level of myogenin in HGF/LIF-treated Mstn^ln/ln^ mice is approximately 3 times higher than in HGF/LIF-treated C57BL/10 mice, since myostatin is not around to exert its repressive influence on MyoD expression, one of the key myogenic factors that regulate myogenin expression [17]. However, as the MyoD expression could have peaked before the time point at which the mice were sacrificed in our study, our MyoD results cannot confirm this. The level of MAFbx and MuRF1 did decrease significantly in HGF/LIF-treated Mstn^ln/ln^ mice in contrast to HGF/LIF-treated C57BL/10 mice. This confirms that MAFbx and MuRF1 expression evidently is dependent on the level of myostatin, albeit not directly, but likely through inhibition of Akt as discussed in the preceding paragraphs. The decrease in structural protein related MuRF1 and ubiquitinylated proteins may explain the modest but significant increase in TA muscle mass and the significant decrease in ubiquitinylated proteins.

We have noted that HGF/LIF treatment is not dependent on damaged myofibers to be effective *in vivo*. This is consistent with the published *in vitro* experiments involving HGF treatment of myoblasts [10], but also implies that treatment with HGF/LIF at the initial dose we gave likely does not depend on a certain level of endogenous co-factors or timing in relation to cellular events. In many ways, the actions of HGF resemble the hypertrophy induced by IGF-1 growth factor, which through its receptor tyrosine kinase is capable of activating the mTOR/p70S6K protein synthesis pathway [28]. At this time, we do not know what the initiation of protein synthesis implies, since it appears to peak at 1 hour post-injection and returns to basic level after 48 hours. It is possible that protein synthesis at this stage merely means expression of MyoD and myostatin, followed by a complicated web of crossing intra-cellular signaling events eventually leading to post-48 hours expression of sarcomeric proteins.

Our results demonstrate a significant effect of HGF/LIF treatment, however, it appears obvious, based on our results from the Mstn^ln/ln^ mice, that treating animals with myostatin inhibiting factors in addition to HGF/LIF may yield an even better effect. It would be reasonable to expect an attenuation of the myostatin-dependent repression of MyoD that is evident even in HGF/LIF-treated animals, and lead to an increase in muscle mass [2].

While we have used normoxic mice for the mechanistic studies and a hypoxia-induced atrophy model for a gain of function through increased muscle mass, the relevance of the latter model for human muscular atrophies and dystrophies can be argued. However, hypoxemia in patients with Duchenne muscular dystrophy (DMD) is well documented [29], and research using animal models of DMD suggests that respiratory insufficiency leads to a chronic hypoxic state in the muscles, which to some extent probably can be attributed to the accompanying cardiomyopathy [30]–[33]. The known and unknown changes in muscle function/molecular signaling is the main reason we decided not to use the DMD *mdx* mouse model for this study as the mechanics of HGF/LIF treatment may very well have been obscured by the *mdx* phenotype, thus defeating the main aim of this study [34]. We believe that the HGF/LIF therapy approach could prove beneficial to many muscular dystrophies and conditions leading to muscular atrophy as long as the primary defect is not affecting the muscle regeneration, which we have demonstrated is the case in some patients with limb-girdle muscular dystrophy type 2A (calpainopathy) [35]. Also, we have recently demonstrated that severely affected patients with limb girdle muscular dystrophy type 2I (fukutin-related protein deficiency) have a significant ongoing regeneration of muscle and increased levels of myostatin and MAFbx and MuRF1 [36], [37]. A compound therapy with HGF/LIF and myostatin inhibitors may attenuate some of the adverse effects of regeneration-mediated myostatin up-regulation and let the regeneration run its due course with an attenuated level of protein degradation. In fact, a premature initiation of the myostatin-mediated protein degradation as presented in this study may in part explain why patients with severe muscular dystrophy and significant ongoing regeneration still experience progressive muscle wasting. The fact that the therapy is independent of the condition, but only depends on the ability to activate satellite cells, the inherent down-stream amplification of growth factor activation signals and the circumvention of immune response issues, makes it attractive seen from a treatment perspective. Further studies of HGF/LIF treatment of dystrophic animals under normoxic conditions will reveal if the beneficial effect of the therapy extends to these animals.

## Supporting Information

Table S1 **Table S1 describes the primer sequences used for qPCR as well as size of amplicon and annealing temperature.**
(DOCX)
