# Supplementary material for: Muscle Atrophy Reversed by Growth Factor Activation of Satellite Cells in a Mouse Muscle Atrophy Model
Source: PLoS One. 2014 Jun 25;9(6):e100594. doi: 10.1371/journal.pone.0100594 (PMC4070942; doi:10.1371/journal.pone.0100594)
Supplement: Table S1 — describes the primer sequences used for qPCR as well as size of amplicon and annealing temperature. (DOCX) [file pone.0100594.s001.docx]

Table S1. Primer sequences used for qPCR

| Target | Forward/Reverse | Amplicon size (bp) | Annealing  temperature (°C) |
| --- | --- | --- | --- |
| Ms_myoD1 | CACATCCTTTTGTTTGTCACTTTCTG/ AGTGGCCTCCGCAAGCT | 72 | 61.5 |
| Ms_myogenin | TTGCTCAGCTCCCTCAACCAGGA/ TGCAGATTGTGGGCGTCTGTAGG | 194 | 63.5 |
| Ms_myostatin | GCCAAGAGCGCCTCCACTCC/  TGGGCTTGCCATCCGCTTGC | 164 | 62.5 |
| Ms_MAFbx | CCATCCTGGATTCCAGAAGATTC/  TCAGGGATGTGAGCTGTGACTTT | 78 | 59 |
| Ms_MURF1 | TACCAAGCCTGTGGTCATCCTG/  ACGGAAACGACCTCCAGACATG | 133 | 66 |
| Reference genes | | | |
| Ms_GAPDH | AATGGGGTGAGGCCGGTGCT/  ATCGGCAGAAGGGGCGGAGA | 125 | 62 |
| Ms_PGK1 | GATGCTTTCCGAGCCTCACTGT/  ACCAGCCTTCTGTGGCAGATTC | 117 | 64.5 |
